# Proposed Diagnostic Criteria, Classification Schema, and Review of Literature of Notochord-Derived Ecchordosis Physaliphora

**DOI:** 10.7759/cureus.547

**Published:** 2016-03-30

**Authors:** Carlito Lagman, Kunal Varshneya, J. Manuel Sarmiento, Alan R Turtz, Rohan V Chitale

**Affiliations:** 1 Department of Neurosurgery, Cooper Neurological Institute, Cooper University Hospital; 2 Center for Neurosurgical Outcomes Research, Maxine Dunitz Neurosurgical Institute, Cedars-Sinai Medical Center; 3 Department of Neurosurgery, Cedars-Sinai Medical Center; 4 Department of Neurological Surgery, Vanderbilt University Medical Center

**Keywords:** ecchordosis physaliphora, chordoma, retroclival lesion, notochordal remnant

## Abstract

Ecchordosis physaliphora (EP) is a benign notochordal remnant derived from ectopic nests found along the craniospinal axis. It typically presents asymptomatically and is diagnosed using classic radiologic features, particularly location, T1-hypointensity, T2-hyperintensity, and lack of enhancement following gadolinium (Gd) contrast administration. Distinguishing EP from its malignant counterpart, chordoma, is of paramount importance, given the aggressive nature of the latter. Advances in imaging and immunohistochemistry have aided in diagnosis to an extent but, to our knowledge, identification of the genetic fingerprint of EP has yet to take place. Further cytological analysis of these lesions in search of a genetic link is warranted. We propose here a set of diagnostic criteria based on features consistently cited in the literature. In this literature review, 23 case reports were identified and collated into a summary of symptomatic cases of ecchordosis physaliphora. An illustrative case report of two patients was also included.

## Introduction and background

Ecchordosis physaliphora (EP) is a benign, congenital lesion representing a notochordal remnant thought to arise by perforation of ectopic nests through the axial skeleton. It occurs particularly at the level of the clivus and sacrum where a convoluted course provides a detour from these cells’ natural embryologic path [1]. Distinguishing EP from its malignant counterpart, chordoma, is of paramount importance. EP is usually asymptomatic, whereas chordomas can present with headache, multiple cranial nerve palsies, and rapid progression to death, despite surgical debulking and radiation therapy [2]. Diagnosis relies on a multimodal approach, taking into consideration the radiologic characteristics and, if made possible, by biopsy, histopathologic, and immunohistochemical features. The radiologic profile of both these lesions provides the most clinical utility, particularly in deciding whether to pursue conservative measures, such as serial imaging for EP, versus surgical resection with postoperative radiation for chordoma. We conducted a systematic review of case reports described in the literature, providing a nearly complete dossier of symptomatic cases of EP, from which we propose diagnostic criteria as well as a clinical classification system for EP, both of which are currently lacking. We believe these two tools can help guide neurosurgeons in the management and prevention of potentially catastrophic events (e.g., sudden death due to subarachnoid hemorrhage) that could occur when one favors a conservative approach with a high-grade EP or mistakenly diagnoses EP in the context of a malignant chordoma [3].

## Review

### Characterization of two asymptomatic EP cases at our institution

**Figure 1:**
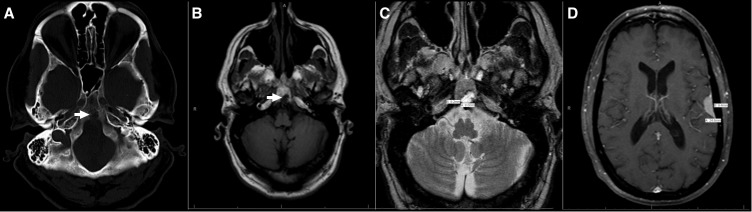
Brain Radiography for Patient 1 (A) Axial CT (bone window);* *(B) T1 Axial MRI; (C) T2 Axial MRI; (D) Axial MRI

A 69-year-old man with a three-month history of hearing loss and a clival lesion identified on an MRI of the internal auditory canal (IAC) presented for neurosurgical consultation. The patient had an occasional mild headache and some mild memory impairment but otherwise no relevant symptoms. Neurologic examination was unremarkable. An axial CT head bone window revealed a low-attenuating lesion with sclerotic margins and a bony stalk within the dorsal aspect of the clivus (Figure 1A). The lobulated mass was hypointense on T1WI (Figure 1B) and hyperintense on T2WI (Figure 1C). Enhancement following contrast administration was not observed. The appearance was most consistent with ecchordosis physaliphora. In addition, two homogenously enhancing meningiomas were noted overlying the inferior aspect of the left parietal lobe (Figure 1D) and the anterolateral aspect of the left temporal lobe (not shown). Approximately five months after initial presentation, the patient suffered a seizure.

**Figure 2:**
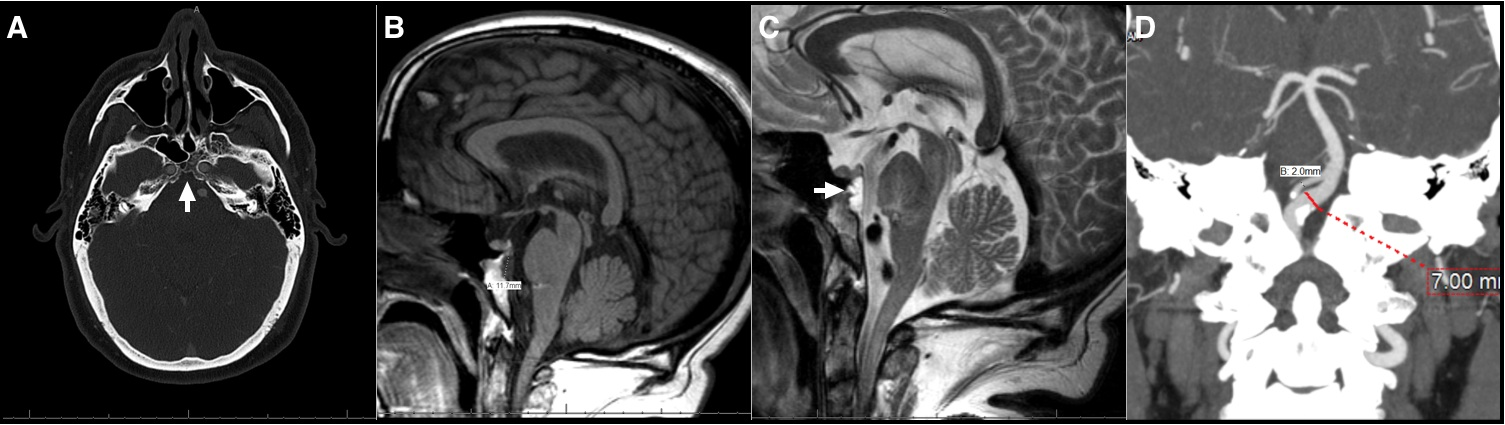
Brain Radiography for Patient 2 (A) Axial CTA (bone window); (B) Sagittal T1 MRI; (C) Sagittal T2 MRI showing EP and pontine telangiectasia; (D) CTA showing fenestrated basilar artery

A 52-year-old man presented for evaluation of multiple anomalies found on a brain MRI. He had no headaches, nausea, or vomiting. Neurologic examination was unremarkable. Prior to consultation, he was experiencing neck and back pain with paresthesias in his arms and legs. Imaging of his lumbar spine showed an L4-5 herniated disc. Imaging of his cervical spine was unremarkable but showed suspicious brain anomalies. Scalloping of the posterior clivus with a possible bony stalk was identified (Figure 2A). Brain MRI showed a T1-hypointense (Figure 2B) and a T2-hyperintense lesion measuring 0.5 cm AP by 1.2 cm transverse at the posterior aspect of the clivus along with possible capillary telangiectasia versus a tiny venous angioma in the pons (Figure 2C). CT angiogram (CTA) with and without contrast and brain MRI were performed to evaluate these lesions. The CTA (Figure 2D) demonstrated dolichoectasia of the left V4 segment (0.6 cm), a fenestrated proximal basilar artery with mild ectasia (0.7 cm). The clival lesions were consistent with a notochordal remnant (e.g., ecchordosis physaliphora); however, a thin-cut MRI was warranted to further evaluate this lesion in an attempt to rule out a more concerning lesion, such as a chordoma, which would require further workup or treatment.

### Methods

A review of the literature was conducted with the use of PubMed (http://www.ncbi.nlm.nih.gov/pubmed/). Search terms “ecchordosis physaliphora” were used and yielded 51 results; dates of publication were between 1982-2016. The titles and abstracts of these publications were screened and only articles containing actual case reports were included. Twenty-four case reports were identified and collated into a summary of symptomatic cases of ecchordosis physaliphora. Full texts of 19 of the 24 English articles (Table 1) were reviewed. We were unable to access full texts of five of the 24 articles but obtained relevant data from the associated abstracts. From the data collected, we proposed diagnostic criteria taking into consideration the clinical, radiologic, histopathologic, and immunohistochemical features.

**Table 1:** Summary of Symptomatic Cases of Ecchordosis Physaliphora *B/l = bilateral. UE = upper extremity​

Reference	Age, Sex	Presentation	CT	T1	T2	Gd	Histopathology	Immunohistochemistry	Management
Choudhri, et al., World Neurosurg, 2014 [1]	63, M	Progressive b/l UE* tremor, intermittent H/A	2.1 cm x 1.8 cm x 1.7 cm pre-pontine mass arising from mid-clivus and extending posteriorly to compress the pons and displace the basilar artery to the left	Hypointense	Hyperintense	No	Lobules of cords and irregular clusters of epithelioid cells set within a rich myxoid background. Frequent physaliphorous cells with cytoplasmic vacuoles were also noted	Brachyury nuclear (+), Ki-67 < 1%	Endoscopic endonasal transclival approach (EETA)
Dias, et al., Clinical Neurol Neurosurg, 2014 [12]	54, F	Severe headache, nuchal rigidity, fever, vomiting, confusion	Cystic lesion inside the sphenoid sinus. Osseous defect in the superior part of the clivus. No bone erosion. CSF fistula	Hypointense	Hyperintense	No	Well-circumscribed aggregate of cells, w/o surrounding tissue invasion or bone involvement. Cells immersed in a myxoid and amphiphilic matrix and numerous, large intracytoplasmic mucin-containing vacuoles	AE1 and AE3 clones, EMA (+), S-100 (-)	Endoscopic endonasal surgery
Kaul, et al., J Neurol Surg, 2013 [3]	52, F	SBM, transclival pseudomeningocele	Well-defined and corticated bony defect in the dorsal wall of the clivus, measuring approximately 6 mm, and a soft tissue mass in the sphenoid sinus	Hypointense	Hyperintense	No	NR	NR	Endoscopic endonasal approach
Bolzoni-Villaret, et al. Laryngoscope, 2014 [28]	51, F	Recurrent CSF leakages	Fluid collection in the right sphenoid sinus, with evident remodeling of the posterior wall. Clival bony defect with a lobulated, non-enhancing mass 12 x 6 mm	NR	Hyperintense	NR	NR	NR	Transphenoidal-transclival endoscopic approach (TTEA)
Krisht, et al., J Neurosurg Pediatr, 2013 [18]	16, F	Diplopia	3.0 x 1.7 x 1.8-cm (“giant ecchordosis physaliphora”), extra-axial epidural mass along the dorsal aspect of the clivus	Intermediate	Hyperintense	No	(unable to access full text)	(unable to access full text)	Transnasal transsphenoidal approach
Yamamoto, et al., Surg Neurol Int, 2013 [17]	20, M	Sudden onset diplopia (cranial nerve VI palsy)	Lesion measuring 22 mm in diameter	Hyperintense	Hyperintense	No	Hypocellular physaliphorous cells with a lobular growth pattern and eosinophilic cytoplasm with vacuolated mucus droplets. Neither mitosis nor dyskaryosis was visible	Cytokeratins (+), MIB-1 “not increased”	Endoscopic endonasal transsphenoidal surgery (ETSS)
Adamek, et al., Neurol Neurochir Pol, 2011 [4]	78, M	Incidental autopsy finding	6-mm gelatinous lesion fixed to the basilar artery on its ventral aspect	N/A	N/A	N/A	NR	NR	S/P myocardial infarction (asymptomatic)
Alkan, et al., Turk Neurosurg, 2009 [5]	22, M	Headache and confusion	(unable to access full text)	Hypointense	Hyperintense	No	(unable to access full text)	(unable to access full text)	Medical therapy
Alli, et al., Skull Base, 2008 [6]	52, F	Left-sided rhinorrhea	Skull base revealed 10-mm focal dissolution of the postero-superior wall of the sphenoid sinus/clivus w/ air-fluid level within the sinus.	Hypointense	Hyperintense	No	NR	NR	Endoscopic intranasal approach
Fracasso, et al., Int J Legal Med, 2008 [7]	48, F	Sudden and unexpected death (SAH)	SAH over the hemispheres. 3.2 x 2.2 x 0.4 cm mass occupied the space above the sella turcica, behind the pituitary stalk. Non-infiltrating.	NR	NR	NR	Cell-poor lobules with large eosinophilic cytoplasm and intracytoplasmic mucus droplets (i.e., physaliphorous cells)	KLI, EMA (+), Vimentin and S-100 (+), MIB-1 (-)	N/A
Miki, et al., Minim Invas Neurosurg, 2008 [8]	59, M	Dizziness, gait disturbance, dementia	Triventricular hydrocephalus. Small tumor with a component resembling bone on the dorsal side of the medial clivus	NR	NR	NR	Meshwork of physaliphorous cells containing numerous vacuoles	NR	ETV + tumor resection
Ling, et al., Otol Neurotol, 2007 [21]	45, M	Sudden left-sided hearing loss w/ non-pulsatile tinnitus	30 x 30 x 14 mm irregularly shaped pre-pontine lesion. Mass effect on the pons, w/ remodeling of dorsal wall of clivus and invagination into (R) sphenoid sinus	Hypointense	Hyperintense	No	Irregular clusters, cords, and interlacing strands of cells dispersed within an abundant myxoid matrix. Isolated cells with enlarged hyperchromatic nuclei were present, but there was no frank cytological atypic	CAM 5.2, CK 19, MNF116, AE1 and AE3, S-100 (+), Ki-67 < 1%	Transpetrosal approach to the CPA
Rotondo, et al., J Neurol Neurosurg Psychiatry, 2007 [22]	47, M	Headache and persistent right-sided facial pain	Subtle stalk-like structure projecting from the dorsal wall of the clivus	Hypointense	Hyperintense	No	Physaliphorous cell nests w/ lobular growth pattern. Eosinophilic cytoplasm; vacuolated with a myxomatous matrix. No mitotic activity or cellular pleomorphism. Pedicle consisted of mature cartilaginous cells	NR	Pre-sigmoidal approach with complete mastoidectomy
Takeyama, et al., Pathology, 2006 [20]	12, M	Left hemiparesis and diplopia	Intradural prepontine mass, measuring 4 cm in diameter, with no bone destruction of the clivus	Hypointense	Hyperintense	No	Physaliphorous cells with abundant intracytoplasmic vacuoles and extracellular pools of mucin	Cytokeratin, EMA, S-100 (+), GFAP (-), MIB-1 < 1%	Resection (unspecified)
Cha, et al., Minim Invasive Neurosurg, 2002 [24]	49, M	Worsening dizziness, headache, and gait instability.	Extra-axial, prepontine mass measuring 1.5 cm. No significant bone destruction or calcification within the mass	Hypointense	NR	Yes	Typical physaliphorous cells with mild to moderate anisonucleosis and a myxoid background. No mitotic activity was identified. Aggregates of small, round lymphocytes were present in the lesion	CAM 5.2, AE1/3, EMA (+), S-100. MIB-1 < 1%	Transmaxillary transclival approach (endoscope-assisted resection)
Toda, et al., J Neurosurg, 1998 [9]	56, F	Headache	Small intradural pre-pontine mass connected to dorsal wall of clivus via a delicate stalk, without bone destruction of the clivus	Hypointense	Hyperintense	No	(unable to access full text)	(unable to access full text)	Lateral suboccipital craniectomy (L)
Ng, et al., Br J Radiol, 1998 [19]	?, M	Hemihypoaesthesia, contralateral hemiparesis	Extradural mass lesion on the dorsal surface of the odontoid process; small bony defect in the adjacent cortex of the odontoid process	Intermediate	Hypointense	No	(unable to access full text)	(unable to access full text)	(unable to access full text)
Rengachary, et al., Neurosurgery, 1997 [23]	34, F	Lower interscapular area pain, radiating to right chest	Cystic lesion at the T8-T9 intervertebral foramen with effacement of the thecal sac	Hypointense	Hyperintense	No	Nodular aggregates of well-circumscribed large vacuolated cells, which featured bland round to oval nuclei with mild anisonucleosis (physaliphorous aliphorous cells of notochordal origin)	CAM 5.2, EMA, S-100 (+). EM: several RER/mitochondria	Thoracic transpedicular exploration w/ gross total resection
Akimoto, et al., No Shinkei Geka, 1996 [10]	51, F	Headache and episode of transient diplopia	Isolated hyperostotic, cystic mass at mid-clivus w/ mild compression of the basilar artery and rostral surface of the pons; small pedicle and dural defect	Hypointense	Hyperintense	No	Scattered physaliphorous cell nests, pedicle consisted of mature cartilaginous cells	MIB (-)	Presigmoid approach with gross total resection
Watanabe, et al., Neurol Med Chirt (Tokyo), 1994 [16]	48, M	(R) hearing loss and face hemianesthesia. Bruns’ nystagmus	Large cystic tumor (schwannoma) (R) CPA in addition to a round, low-density mass in the clival bone marrow w/ small defect of the dorsal clival cortex	Hypointense	Hyperintense	No	Physaliphorous cells with variable vacuoles, which were neither atypical nor pleomorphic. No vascular component was found	EMA (+), S-100 (+), keratin (+)	Resection (unspecified)
Macdonald, et al., Neurosurgery, 1990 [2]	66, F	Two-year Hx of CSF rhinorrhea	Mass in the posterior wall of the right sphenoid sinus. Bony defect w/o destruction in the posterior wall of sphenoid sinus into pre-pontine cistern	Hyperintense	Hyperintense	No	Physaliphorous cells arranged in several lobules surrounded by fine connective tissue septa, abundant intracytoplasmic vacuoles (Alcian blue and PAS positive). Extracellular mucin was also observed	Vimentin (+), cytokeratin (+), and S-100 (+), CEA (-), GFAP (-)	Sublabial midline rhinoseptal transsphenoidal approach
Stam FC, Kamphorst W, Eur Neurol, 1982 [11]	75, M	SAH, sudden death	Prepontine	N/A	N/A	N/A	(unable to access full text)	(unable to access full text)	n/a
Filis, et al., Journal of Clinical Neuroscience, 2016 [26]	44, F	Chronic headaches	Cystic mass in pre-pontine cistern	Hypotintense	Hyperintense	N/A	Fragments of neoplasm composed of stellate and polygonal cells with foamy or eosinophilic cytoplasm in an abundant myxoid stroma	Mitotic index (Ki - 67) < 1%, positivity to epithelial membrane antigen and S-100 stain. Brachyury immunostaining confirmed the tumor to be of notochordal origin	Left frontal craniotomy

We went one step further by developing a classification system based on the hospital course and clinical outcomes described in the cases of symptomatic EP in our review with the aim of providing neurosurgeons and neurologists with a grading system that would allow one to rapidly make decisions using only clinical and radiologic features.

### Diagnostic criteria

The histopathologic analysis demonstrates the sine qua non of EP diagnosis, that is, the physaliphorous cells, which were immersed in a myxoid amphophilic matrix and characterized by large mucin-containing intracytoplasmic vacuoles [12]. Other histologic features often described were the absence of mitoses, which corresponded to a low proliferative index and benign nature, as well as the lack of necrosis, sparse pleomorphism, and hypocellularity (Table 2).

**Table 2:** Proposed Diagnostic Criteria for Ecchordosis Physaliphora

Clinical Features
< 6 cm^3 ^(typical) > 6 cm^3 ^(giant EP) CT may feature bony stalk/pedicle (pathologic hallmark) with focal bone destruction T1-hypointense T2-hyperintense Gd non-enhancing
Histopathological Features
Physaliphorous cells characterized by large mucin-containing intracytoplasmic vacuoles Hypocellularity Sparse pleomorphic Absence of mitoses
Immunohistochemical Features
(+) Cytokeratins (e.g., AE1 and AE3), EMA, S-100, galectin-3 (+) Brachyury staining (-) GFAP MIB-1/Ki67 < 1%

Immunohistochemistry provides yet another means by which to increase the positive predictive value of the diagnosis of EP, albeit far from definitive. Positive staining for cytokeratins (e.g., AE1 and AE3), epithelial membrane antigen (EMA), S-100, galectin-3, and brachyury were consistent with notochordal remnants (i.e., both EP and chordoma), as were negative staining for GFAP, CEA, and 5’-nucleotidase. 5’ nucleotidase staining was recognized as early as 1984, by Bottles and Beckstead, as particularly characteristic of chordomas [13]. Lastly, the MIB-1 index and Ki67 were commonly quoted to be less than 1% in cases of EP.

To date, there is no published grading system or guidelines for the management of EP. Chihara, et al. recently proposed a new classification system based on MRI FIESTA (Fast Imaging Employing Steady-state Acquisition) (details found in the conclusion). FIESTA sequences provide a strong signal in tissues with high T2: T1 ratios, such as cerebrospinal fluid (CSF), and high spatial resolution with submillimeter section thickness allowing for reconstruction of multiplanar images. Therefore, steady-state free precession (SSFP) sequences are capable of depicting tiny intracranial lesions in contrast to the traditional MR techniques [14]. Advances in MRI, such as FIESTA, provide clinicians with yet another tool to help identify these lesions much earlier and with greater precision with respect to progression in size. It is worth noting that in the papers by Chihara, et al. and Özgür, et al., an additional combined 26 cases of classical EP (Chihara, n = 17 and Özgür, n = 11) were identified. However, as these articles were not intended as case reports, we were unable to extract sufficient data to include in our review [14-15]. Nevertheless, we attempt to integrate this classification system while codifying the data we have gathered through our review of literature into a new classification schema, which has the potential to guide management. Based on our review of symptomatic cases, all fall within the range of Grade III-V, thus, warranting surgical intervention or at least biopsy as these cases likely represent transformation to malignant chordoma. All but five cases were managed surgically with evolving techniques, details of which are beyond the scope of this review. Of the five patients, no data regarding operative technique was available due to sudden death (n = 3), management via medical therapy (n = 1), and inability to access full text (n = 1). 

### Classification schema

The lack of symptomology is classically attributed to EP; however, the case reports we reviewed were all symptomatic cases of EP. EP is typically found incidentally, either at autopsy or on imaging sought for investigation of symptoms unrelated to the identified EP (i.e., incidental finding). We found two cases of EP with associated vestibular schwannoma. Özgür, et al. identified 11 cases of classical EP, with one patient presenting with concurrent schwannoma. Watanabe, et al. also described a case in which a patient presented with right hearing disturbance and reduction in sensation in the right side of his face due to a large cystic schwannoma [14, 16]. 

As far as radiologic features, the three major criteria we identified were T1-hypointensity, T2-hyperintensity, and lack of enhancement with gadolinium (contrast) administration. These three features were the most consistently cited characteristics that allowed one to make a diagnosis of EP based on imaging alone (Table 2). We identified two cases that did not meet these three radiologic criteria but were diagnosed as EP, taking into account the histopathologic and immunohistochemical features. Both Yamamoto, et al. and Macdonald, et al. describe cases of EP, which displayed T1-hyperintensity, T2-hyperintensity, and lack of enhancement with contrast. Histopathological analysis of both cases showed the classic physaliphorous cells. Immunohistochemical analysis also showed characteristic features, which are discussed later. Two other cases are notable in that they demonstrated intermediate enhancement on T1 [2, 17]. Krisht, et al. and Ng, et al. both presented cases of extradural EP with intermediate intensity on T1WI. The former also described a “giant” EP (3.0 x 1.7 x 1.8 cm) [18-19]. There is currently no published consensus regarding the definition of giant EP. In 2006, Takeyama, et al. published the first case report of EP (4 cm in diameter) that satisfies this metric; however, they do not explicitly call this a “giant” EP [20]. It was not until 2007 when Ling, et al. published the first case report of a giant EP, where the word “giant” explicitly appears in both title and text. That particular mass measured 30 x 30 x 14 mm [21]. The most recent case of a giant EP in which the EP measured 2.1 cm x 1.8 cm x 1.7 cm (6.426 cm^3^) was described by Choudhri, et al. Therefore, the literature suggests that anything greater than 3 cm in diameter or 6 cm^3^ in volume is consistent with a so-called “giant” EP, and we have included this in the radiologic features under our proposed diagnostic criteria [1]. Other features of EP include the location and the associated bony stalk or pedicle. As far as location, EP is classically intradural and near the midline with respect to the craniospinal axis; however, it can be located in any combination of the extradural, subdural, and subarachnoid spaces [7, 17, 22]. EP in the subarachnoid or subdural space is typically connected by a thin pedicle or bony stalk, which is often described, but not often visualized, as an osseous stalk-like projection connecting the lesion (EP) to the dorsal aspect of the clivus; it is considered the pathological hallmark of EP by some authors [12]. In our opinion, Rotondo, et al., in their case report of a symptomatic EP, demonstrated the most distinct and classic image of the stalk-like projection. Focal bone erosion and even destruction are other features we came across on multiple occasions, which has been suggested to be attributable to a higher grade EP (Table 3). 

**Table 3:** Proposed Classification System for Ecchordosis Physaliphora with Proposed Management *Stage IV and V represents malignant transformation from EP to chordoma. Enhancement is observed, implying a higher grade. CN palsy seen in retroclival EP

Grade I	Asymptomatic, classic radiologic features without stalk	Serial MRI	EP likely dx; observe
Grade II	Asymptomatic, classic radiologic features with stalk	Serial MRI	EP most likely dx; observe
Grade III	Symptomatic, classic radiologic features with stalk, < 6 cm^3^,	Biopsy or resection	EP equally as likely as CD; further investigation warranted
Grade IV*	Symptomatic, bone erosion and/or Gd-enhancement, > 6 cm^3^	Surgical resection	Symptomatic EP; resection warranted to alleviate symptomology
Grade V*	CN palsy, bone destruction and Gd-enhancement, T1-hyperintense	Surgical resection	EP transition to CD; resection warranted to prevent complications

### Discussion

Rengachary et al. compiled a review of studies that described the evolution of the perspectives on ecchordosis physaliphora. Herbert Luschka first described small, transparent, jelly-like nodules projecting into the prepontine area from the middle of the clivus, which likely represented the first case of either chordoma or ecchordosis physaliphora as we know it today [23]. In 1857, Rudolf Virchow examined a similar lesion under the microscope. According to Rengachary, et al., Virchow mistakenly identified the origin of this lesion as chondral and thought that it represented a degenerative process affecting the spheno-occipital synchondrosis, thus, designating the condition as ecchondrosis physaliphora (from the Greek, physalis meaning “bubble”) [23]. In 1858, Friedrich von Müller suggested a notochordal origin. In 1898, Ribbert provided experimental evidence to support Müller’s notion by puncturing the intervertebral discs of rabbits, creating iatrogenic herniated nucleus pulposus, and allowing for the proliferation of notochordal rests along the craniospinal axis, thus, simulating the evolution of EP. Upon histological analysis one year later, Ribbert found that the tissue resembled the soft gelatinous nodules noted at the clivus and subsequently coined the term "ecchordosis physaliphora", which implied notochordal rather than chondral origin. In addition, Ribbert described the cyst wall as consisting of layers of fibroblasts within a collagenous matrix containing occasional chronic inflammatory lymphocytes and plasma cells [23]. Although not explicitly stated, what Ribbert had likely also observed was a lack of mitoses, characteristic of EP and reflective of the low proliferative index, which explains the typically asymptomatic presentation. In fact, this mass is often only identified on autopsy (0.5-2.0%), or incidentally, with likely unrelated symptoms, such as in the patients we present in this case report [1]. Dr. Rengachery's work in assimilating these studies was imperative in establishing the diagnostic criteria in the early stages of ecchordosis physaliphora discovery.

In contrast, chordoma often presents with a headache and multiple cranial nerve palsies due to mass effect on the brainstem. Computed tomography (bone window) may demonstrate a bony stalk or pedicle and/or focal bone destruction along the posterior aspect of the clivus. MRI displays an intradural mass with T1-hypointensity, T2-hyperintensity, and gadolinium (Gd) non-enhancement. T1- and T2-intensities are due to the lack of fat and abundance of mucin, respectively, whereas sparse vascularity precludes enhancement. Chordomas, on the other hand, are often extradural, displaying intratumoral calcification, both T1- and T2-hyperintense, and enhance upon administration of Gd due to increased vascularity and disruption of the blood-brain-barrier, a feature common to most malignant lesions [24]. Imaging alone has the potential to guide management of these clival lesions. Asymptomatic EP is managed by serial imaging, often remaining stable over years, whereas chordomas display progression in as little as one year by way of either mass effect or metastasis [21]. 

Symptomatic EP warrants resection, via evolving surgical techniques, as made evident by our review of the literature. Unlike the conservative management of asymptomatic EP, chordomas require a more aggressive approach (i.e., gross total resection with postoperative radiation therapy). The response of EP to radiation therapy (e.g., Gamma Knife) has not been described in the literature. Histologically, EP and chordoma are indistinguishable, owing to similar embryologic derivation. Both demonstrate large cells characterized by copious intracytoplasmic vacuoles and extracellular pools of mucin, which resemble bubbles, hence, the term "physaliphorous". Immunohistochemical methods have aided in demonstrating the aforementioned common notochordal origin of both EP and chordoma, allowing differentiation from other masses, such as neuroenteric cysts, but have provided little in the way of distinguishing EP from chordoma. Despite similar staining characteristics (cytokeratin, EMA, S-100, galectin, and brachyury positivity and GFAP negativity), the literature does describe subtle features, which favor the diagnosis of EP (e.g., a lower Ki-67 index, a very low MIB count, and absence of mitosis) [25-26]. Furthermore, electron microscopy of EP features unique rough endoplasmic reticulum (RER) mitochondrial complexes, intracytoplasmic invaginations, numerous intracytoplasmic vacuoles, and plasma membranes with pinocytotic vesicles and cell junctions. However, EM is less commonly cited as being integral to the diagnosis of EP.

## Conclusions

The two patients described in this literature review presented with classic features of EP (asymptomatic with the characteristic radiologic profile). Based on our proposed classification system, both tumors fall within the range of Grade I-III EP. Advances in imaging and immunohistochemistry have aided in diagnosis to an extent but, to our knowledge, identification of the genetic fingerprint of EP has yet to take place. Thus, further cytological analysis of these lesions in search of a genetic link is warranted, as is the call for reports of EP with other incidental findings. The diagnostic criteria we propose is based on features consistently cited in the literature; however, it is glaringly obvious that in order to meet histological and immunohistochemical criteria, one must obtain a tissue sample via biopsy. Although we reviewed many case reports that described novel techniques of resection, analysis of tissue was often performed after near-total or gross resection of the masses. We did not come across any case report describing biopsy undertaken without subsequent resection. It is conceivable that the benefit of biopsy of Grade III EP (based on our proposed classification system) with subsequent histopathologic and immunohistochemical confirmation outweighs the risks of complete resection and may obviate the need for serial MRI. Chihara, et al. recently proposed a new classification system of EP based strictly on radiologic characteristics identified using contrast-enhanced Fast Imaging Employing Steady-State Acquisition MR imaging (FIESTA). Chihara, et al. separated dorsal surface lesions into classical EP and possible EP. Classical EP was further subdivided into type A: hyperintense excrescence (cyst-like component) on the dorsal surface of the clivus and type B: hyperintense excrescence, plus a hyperintense lesion within the clivus. Possible EP was further subdivided into incomplete EP characterized by a T2-hypointense protrusion of the clivus and an EP variant characterized by hyperintense lesions within only the clivus [7]. Our classification system attempts to extrapolate gradation of these lesions in the context of clinical presentation in order to help guide management.
